# Association between Optic Neuritis and Inflammatory Bowel Disease: A Population-Based Study

**DOI:** 10.3390/jcm10040688

**Published:** 2021-02-10

**Authors:** Yun-Hsiu Hsieh, Chi-Hsiang Chung, Chien-An Sun, Po-Huang Chen, Yi-Hao Chen, Chang-Min Liang, Jiann-Torng Chen, Wu-Chien Chien, Ching-Long Chen

**Affiliations:** 1 Department of Ophthalmology, Tri-Service General Hospital, National Defense Medical Center, Taipei City 11490, Taiwan; vv7788442200@gmail.com (Y.-H.H.); doc30879@ndmctsgh.edu.tw (Y.-H.C.); doc30875@yahoo.com.tw (C.-M.L.); jt66chen@gmail.com (J.-T.C.); 2 School of Public Health, National Defense Medical Center, Taipei City 11490, Taiwan; g694810042@gmail.com; 3 Department of Medical Research, Tri-Service General Hospital, National Defense Medical Center, Taipei City 11490, Taiwan; 4 Taiwanese Injury Prevention and Safety Promotion Association, Taipei City 11490, Taiwan; 5 Department of Public Health, College of Medicine, Fu-Jen Catholic University, New Taipei City 242062, Taiwan; 040866@mail.fju.edu.tw; 6 Big Data Research Center, College of Medicine, Fu-Jen Catholic University, New Taipei City 242062, Taiwan; 7 Department of Internal Medicine, Tri-Service General Hospital, National Defense Medical Center, Taipei City 11490, Taiwan; chenpohuang@hotmail.com; 8 Graduate Institute of Life Sciences, National Defense Medical Center, Taipei City 11490, Taiwan

**Keywords:** Crohn’s disease, extraintestinal manifestation, inflammatory bowel disease, optic neuritis, ocular manifestation, ulcerative colitis

## Abstract

Extraintestinal manifestations are common in patients with inflammatory bowel disease (IBD), and optic neuritis (ON) is a rare but severe one. This study aimed to evaluate possible factors associated with ON in patients with IBD. Adult patients with IBD who were not with concomitant ON on the index date identified from the Taiwan National Health Insurance Research Database (NHIRD) from the years 2000 to 2013 were included. A four-fold matched group was selected using age, sex and year of index date for comparison. All the patients were followed up until the development of ON or the end of the study period. Data of included patients were extracted and analyzed statistically. The mean follow-up time for all patients was 7.13 ± 5.21 years. At the study period conclusion, eight (0.18%) and five (0.003%) patients with and without IBD, respectively, had developed ON (*p* = 0.001). Adjusted HRs showed that patients with IBD aged between 30 and 39 years, with comorbidities including neuromyelitis optica (NMO), acute disseminated encephalomyelitis (ADEM), systemic lupus erythematosus (SLE) and with a higher Charlson Comorbidity Index, had a significantly higher risk of developing ON (all *p* < 0.005). Among the eight IBD patients who developed ON, only one patient was diagnosed with Crohn’s disease, the male gender was slightly dominant, and two (25%) patients received antitumor necrosis factor α (anti-TNF α) treatment for IBD. Patients with IBD have a higher risk of developing ON compared to patients without IBD. ON occurs more frequently in IBD patients aged between 30 and 39 years, with comorbidities including NMO, ADEM and SLE. Other factors besides anti-TNF α treatment for IBD are more likely associated with the development of ON.

## 1. Introduction

Inflammatory bowel disease (IBD) comprises two main distinctive diseases, ulcerative colitis (UC) and Crohn’s disease (CD), and is a chronic, idiopathic inflammatory condition occurring in the gastrointestinal (GI) tract [1]. IBD primarily affects people around 15–35 years old, regardless of sex, and may persist nearly throughout life [2]. UC usually affects the colon in an uninterrupted pattern, whereas CD may affect any region of the intestine [3]. The major complaints of patients with IBD are colonic symptoms due to colonic tissue damage caused by gastrointestinal inflammation; however, 6% to 47% of IBD patients are reported to present extraintestinal manifestation (EIM), depending on the definitions applied across the studies [4,5,6], which means experiencing symptoms outside the GI tract such as in joints, mouth, skin, liver and eyes [7,8].

Optic neuritis (ON) is a demyelinating inflammation or degeneration of the optic nerve, including the optic nerve head, optic nerve sheath and optic nerve itself, leading to complete or partial visual impairment due to the swelling and destruction of the myelin sheath covering the optic nerve [9,10]. The incidence of ocular IBD EIM ranged from 1.0% to 43% in previous studies [11,12,13], and ON may present in up to 4% of these IBD patients [11,14]. Although rare, due to the severe impact on the quality of life, it is important to understand the features of ON within the disease course of IBD, including the pathologic mechanisms and risk factors, to avoid irreversible loss of vision.

The etiology of ON in IBD patients remains a matter of controversy. ON can be seen as an isolated disease, a sign of autoimmune disease like IBD, a manifestation of neurologic disease or an early sign of demyelination disease, such as multiple sclerosis (MS) [15,16,17,18]. In particular, the link with IBD has been mentioned for decades. For example, a retrospective cohort study revealed that MS and ON occur more commonly among patients with IBD than among non-IBD patients [19], making clarifying the etiology of ON in patients with IBD more difficult. In addition, secondary antitumor necrosis factor α (anti-TNF α)-induced or anti-TNF α-associated ON has been reported in association with the wider use of anti-TNF α therapy for a variety of autoimmune diseases, including IBD and MS [20,21,22,23,24]. All of the above-mentioned factors expand the complexity and possible underlying mechanisms related to the occurrence of ON in patients with IBD.

This study aimed to evaluate the possible factors associated with the incidence of ON in patients with IBD. Our ultimate goal was to increase awareness of ON in patients with IBD and provide useful information for all the physicians in the multidisciplinary care team treating this rare condition.

## 2. Methods

### 2.1. Data Source

Medical data were extracted from the deidentified National Health Insurance Research Database (NHIRD) from 2000 to 2013. More than 25 million people are eligible for participation in the National Health Insurance (NHI) program, including up to 99.91% of the residents of Taiwan and non-Taiwanese residents living in Taiwan currently. The NHIRD contains registration files and original claims data for reimbursement. For the present study, age, sex, index year, clinical visits, medications and diagnostic codes were extracted and analyzed. The International Classification of Disease, Ninth Revision, Clinical Modification (ICD-9-CM) was used to define the diagnostic codes. The ICD-9-CM codes used in the present study are listed in Table S1. Anti-TNF α use was defined as at least 3 times the prescription records of adalimumab (Humira; AbbVie Inc., North Chicago, IL, USA, drug code: KC01039271) and infliximab (Remicade; Janssen Biotech, Inc., Malvern, PA, USA; drug code: KC00980255, Remsima; Celltrion Inc., Incheon, Korea, drug code: KC01035255) after the diagnosis of IBD and before the diagnosis of ON, given that the NHI approved the use of these two drugs only for the treatment of IBD during the study period.

### 2.2. Ethical Considerations

The study protocol was reviewed and approved by the Institutional Review Board of Tri-Service General Hospital (TSGH IRB No. A202005062). Because the data from NHI were deidentified, signed informed consent of included patients was waived.

### 2.3. Patient Selection

**Figure 1:**
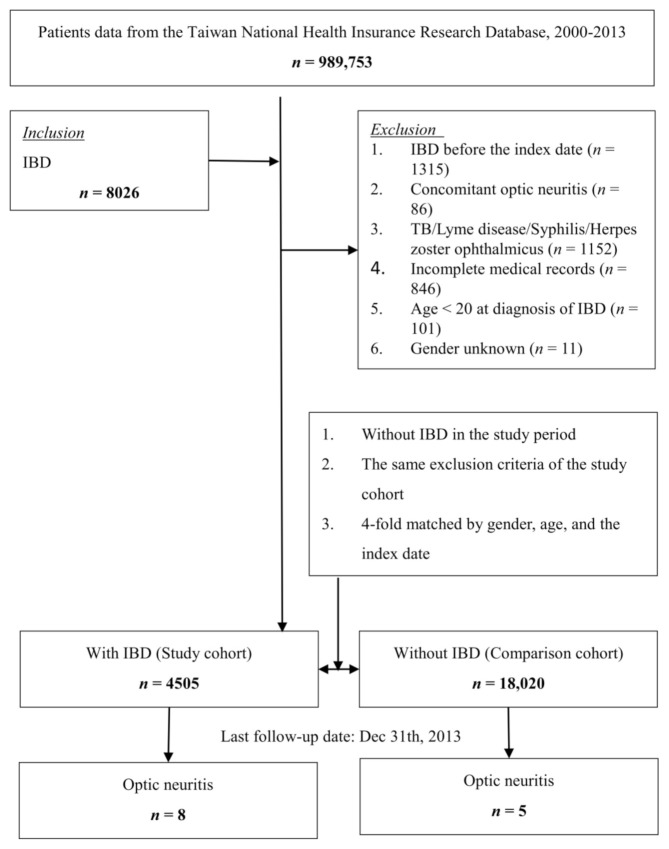
Flowchart of this study.

All included patients were at least 20 years old at diagnosis of IBD, had records of outpatient or inpatient visits with a diagnosis of IBD at least 3 times during the study period and not limited to the main diagnosis. Patients with tuberculosis, Lyme disease, syphilis and Herpes zoster ophthalmicus from before 1 year to after 1 year of the index date were excluded. In addition, patients who were documented as having IBD before 1 January 2000 or had incomplete medical records, defined as the insurance status being incomplete or the given codes unrelated or incorrect, were also excluded. A group of patients who were four-fold matched with the IBD group was selected as the control, using age (each 5-year span), sex and year of the index date for matching. Patients were followed until the incidence of ON in the records of outpatient or inpatient visits, or until the end of the study period (31 December 2013). Figure 1 shows a flowchart of the patient selection.

### 2.4. Statistical Analysis

Pearson’s chi-square test and Fisher’s exact test were used to evaluate the differences in categorical variables, including sex and age group. After adjusting for confounding variables, univariate and multivariate Cox regression analyses were employed to evaluate the adjusted hazard ratios for the influence (odds) of analyzed variables on developing ON. Kaplan–Meier analysis was performed to estimate the development of ON in these two cohorts. All statistical analyses were performed using SPSS software (version 22.0; SPSS Inc., Chicago, IL, USA). Statistical significance was defined as *p* < 0.05.

## 3. Results

Table 1 shows the demographic and clinical characteristics of the enrolled patients at baseline. A total of 22,525 patients were enrolled, including 4505 patients with IBD and 18,020 age-, sex- and index-year-matched patients. The mean ages of patients with and without IBD were 55.36 ± 17.45 and 55.13 ± 17.53 years, respectively. The mean follow-up time was 7.13 ± 5.21 (range: 0.01 to 13.99) years for all patients.

For comorbidities, higher proportions of MS, neuromyelitis optica (NMO), acute disseminated encephalomyelitis (ADEM), sarcoidosis, systemic lupus erythematosus (SLE), Behçet syndrome, antiphospholipid antibody syndrome, granulomatosis with polyangiitis (GPA) or Sicca syndrome were found in patients with IBD (all *p* < 0.001), compared to those without IBD. Patients with IBD had higher Charlson Comorbidity Index (CCI) values than patients without IBD (*p* < 0.001). In patients with IBD, 987 (21.91%) were treated with anti-TNF α drugs.

**Figure 2:**
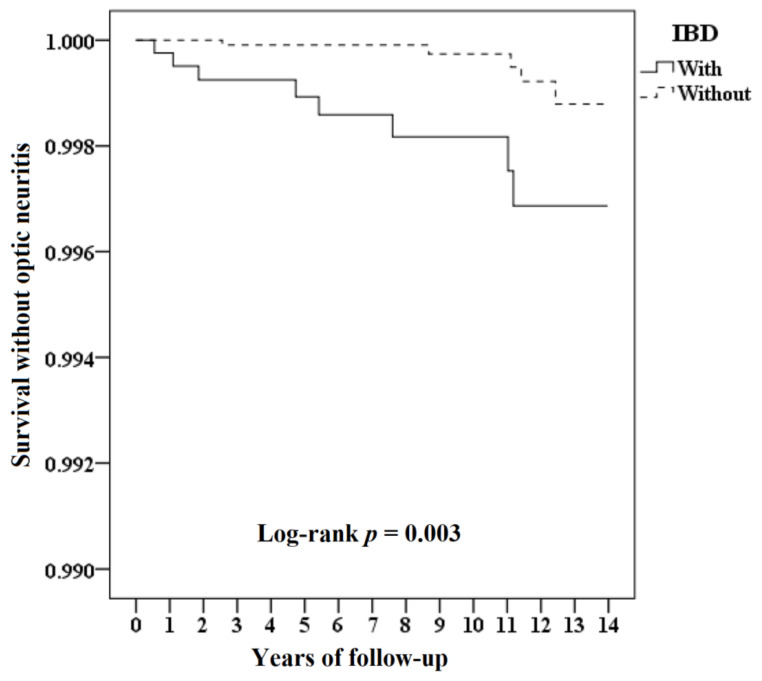
Kaplan–Meier curve for optic neuritis, stratified by IBD with the log-rank test.

At the study conclusion, eight (0.18%) and five (0.003%) patients had developed ON in the patients with and without IBD groups, respectively, with a significant difference in prevalence shown between the two groups (*p* = 0.001). The mean time for ON to develop for patients with IBD was 5.43 ± 4.23 years and 9.24 ± 3.98 years for patients without IBD. Results of the Kaplan–Meier analysis for the cumulative risk of developing ON in patients with IBD and patients without IBD are shown in Figure 2. Patients with IBD had significantly higher odds of developing ON compared to patients without IBD, with log-rank test *p* = 0.003. Differences in risk between the two groups were significant in each year of the total 14 years of follow-up (all *p* < 0.016). Adjusted HRs showed that patients with IBD aged between 30 and 39 years, with comorbidities including NMO, ADEM and SLE, and with a higher CCI had a significantly higher risk of developing optic neuritis (Table 2).

The results of subgroup analysis comparing patients with and without IBD are shown in Table 3. The overall incidence of ON was 20.25 per 100,000 person-years in patients with IBD and 4.27 per 100,000 person-years in patients without IBD. Patients with IBD had an increased risk of developing ON regardless of sex. The adjusted HR was 4.503 in males and 3.831 in females (*p* < 0.001). IBD patients in the 30 to 39 and 50 to 59 years age groups were independently associated with a significantly increased risk of developing ON than patients without IBD, with an adjusted HR of 5.096 in the 30–39 years age group and 3.642 in the 50–59 years age group (*p* < 0.001). For comorbidities, IBD patients with MS, NMO, ADEM and SLE had significantly higher risks for developing ON, with adjusted HRs of 3.759, 3.202, 3.306 and 3.896, respectively (all *p* ≤ 0.005). IBD patients without Behçet syndrome, antiphospholipid antibody syndrome or GPA also had a significantly higher risk of developing ON (adjusted HRs all 4.211, *p* = 0.001).

Detailed information of the eight patients in the IBD group who developed ON is listed in Table 4. Only one patient was diagnosed with Crohn’s disease, and the male gender was slightly dominant, with five males among the eight patients. Only two patients received anti-TNF α treatment for IBD. The time for ON to develop after diagnosis of IBD was diverse, ranging from within 1 year to 11.18 years.

## 4. Discussion

In this population-based cohort study, 4505 patients with IBD were identified from a million-level database and followed for a mean follow-up of 7 years. Only eight (0.18%) patients with IBD developed ON but still had a significantly higher rate than patients without IBD. The incidence of ON in patients with IBD was 20.25 per 100,000 person-years and over four-fold higher than that in patients without IBD. Concurrence of other autoimmune diseases was significantly more frequent in patients with IBD compared to patients without IBD. Within the eight patients in the IBD group who developed ON, two patients received anti-TNF α treatment for IBD, which may suggest that other factors besides the use of anti-TNF α agents are more likely associated with ON.

The prevalence and incidence of ophthalmic manifestations in patients with IBD vary across studies [25]. The reported prevalence of ophthalmic manifestations in IBD in surveys and medical chart review studies ranged from 2% to 5% [26,27,28] and was even lower (1% to 2%) in a population-based study [4,19]. Our result of 0.18% was higher than that in a population-based study conducted in 2005, in which around 0.11% was reported [19]. We used the claims data based on the ICD-9-CM of this ocular finding coded by the ophthalmologists after evaluation. Felekis et al. [11] found that ophthalmic manifestations were significantly more prevalent after a thorough evaluation by the ophthalmologist is performed, which may be a possible reason. However, due to the small numbers (only 13 patients, 8 and 5 patients with and without IBD, respectively, who had developed ON in this large cohort), the significance revealed by the Kaplan–Meier analysis must be explained carefully to avoid overstating and misleading.

Adverse effects of anti-TNF α have been discussed intensively as the possible etiology of new-onset demyelination diseases in patients with IBD. For ON, several case reports have indicated that this may be due to the onset time being close to the start of the anti-TNF α treatment, and the condition was reversed in some cases after discontinuing the use of anti-TNF α agent [22,23,24,29,30]. The issue remains inconclusive and unresolved. Gupta et al. performed a retrospective cohort study and a retrospective cross-sectional study using the data of the era before anti-TNF α treatment (1988 to 1997), and the results demonstrated that regardless of anti-TNF α use, patients with IBD were more likely to have a higher incidence of MS, demyelination and ON compared with a matched control. In a recent retrospective multicenter study of a large database and a comprehensive literature review, Alexandre et al. [30] identified 26 cases of optic neuritis from the literature and the European Crohn’s and Colitis Organisation’s Collaborative Network for Exceptionally Rare case reports (CONFER) database. Sixteen patients were under anti-TNF α treatment, and 10 patients were without anti-TNF-α treatment. The authors concluded that the causal association between anti-TNF α and the onset of ON was still uncertain. These results are consistent with our results, albeit probably not all ON occurring in patients with IBD are related to the use of anti-TNF α agents.

It is well known that in patients with IBD, comorbid IBD usually coexists with other autoimmune diseases. For example, a high association has been reported between IBD and MS [15,16,17,18,19]. ON can be seen as the first presenting sign of MS, and the cumulative probability of developing MS after onset of ON within 15 years is 50% [31]. ON also is one of the EIMs of IBD, indicating the possibility that ON may be caused by the underlying predisposition of IBD [32]. However, the results of the present study still cannot draw a clear relationship between MS, IBD, ON and other autoimmune and/or demyelinating diseases.

A major strength of this study is the use of a nationwide Taiwanese population sample. This design reduced selection bias and made the results applicable to the general population of Taiwan and accurate for the evaluation of a rare condition like ON. However, the results are limited by the uniform ethnic background. Two studies, both conducted in the United States, evaluated whether there is any difference between patients with IBD who live in the United States with different ethnic backgrounds. A cohort study conducted by Kocher et al. reported that Asians were more likely to have ocular manifestations compared to whites (3.4% versus 0.7%, *p* = 0.022) [33]; however, a systematic review of 47 studies indicated that no major differences were seen in EIM among races and ethnic groups [34]. To the best of our knowledge, no data about the incidence of ON in different ethnic groups are reported, and the impact of genetic background needs to be explored in the future.

There are still some other limitations of the present study. The NHIRD database does not provide laboratory or clinical information such as MRI findings for the classification of disease severity, which might also play a role in developing ON [35]. For the same reason, the accuracy of diagnosis cannot be reconfirmed in this study. We did not extract all the medications used for IBD, which may miss some possible causes of ON. Furthermore, the impacts of other ocular manifestations, including episcleritis and scleritis in the incidence of ON in patients with IBD, were not analyzed since they are rare. Studies for further evaluation of the association should be conducted in the future.

In conclusion, patients with IBD have a higher risk of developing ON compared to patients without IBD. Because IBD has been known to be related to many neurological, immunological and ophthalmic conditions, a multidisciplinary care team may be necessary for diagnosis and to discuss appropriate programs of treatment and follow-up. Early and routine screening of visual function in patients with IBD may be important, especially in patients with a higher risk of ON.

## Figures and Tables

**Table 1:** Patients’ demographic and clinical characteristics at baseline.

	With IBD		Without IBD		*p*
Variables	*n*	%	*n*	%	
**Total**	4505	20.00	18,020	80.00	
**Gender**					0.999
Male	2427	53.87	9708	53.87	
Female	2078	46.13	8312	46.13	
**Age (years) ^a^**	55.36 ± 17.45		55.13 ± 17.53		0.432
**Age groups (years)**					0.999
20–29	448	9.94	1792	9.94	
30–39	655	14.54	2620	14.54	
40–49	675	14.98	2700	14.98	
50–59	637	14.14	2548	14.14	
≥60	2090	46.39	8360	46.39	
**Multiple sclerosis**					<0.001 *
Without	4379	97.20	17,918	99.43	
With	126	2.80	102	0.57	
**Neuromyelitis optica**					<0.001 *
Without	4407	97.82	17,978	99.77	
With	98	2.18	42	0.23	
**Acute disseminated encephalomyelitis**					<0.001 *
Without	4392	97.49	17,953	99.63	
With	113	2.51	67	0.37	
**Sarcoidosis**					<0.001 *
Without	4427	98.27	17,988	99.82	
With	78	1.73	32	0.18	
**SLE**					<0.001 *
Without	4230	93.90	17,789	98.72	
With	275	6.10	231	1.28	
**Behçet syndrome**					<0.001 *
Without	4472	99.27	18,008	99.93	
With	33	0.73	12	0.07	
**Antiphospholipid antibody syndrome**					<0.001 *
Without	4486	99.58	18,017	99.98	
With	19	0.42	3	0.02	
**Granulomatosis with polyangiitis**					<0.001 *
Without	4477	99.38	18,013	99.96	
With	28	0.62	7	0.04	
**Sicca syndrome**					<0.001 *
Without	4307	95.60	17,942	99.57	
With	198	4.40	78	0.43	
**CCI ^a^**	1.76 ± 1.98		1.55 ± 1.63		<0.001 *
**Anti-TNF use**					
Without	3518	78.09	NA	NA	
With	987	21.91	NA	NA	

* indicates a significant difference between the 2 groups with/without IBD, *p* < 0.05. ^a^ presented as mean ± SD. *CCI:* Charlson comorbidity index, IBD: inflammatory bowel disease, NA: not available, NTD: new Taiwan dollar, SLE: systemic lupus *erythematosus,* TNF: tumor necrosis factor.

**Table 2:** Risk analysis for optic neuritis.

Variables	Crude HR (95% CI)	*p*	Adjusted HR (95% CI)	*p*
**IBD**				
Without	Reference		Reference	
With	4.020 (1.366–10.257)	0.003 *	4.211 (1.352–12.978)	0.001 *
**Gender**				
Male	1.168 (0.482–3.279)	0.812	1.053 (0.351–3.240)	0.873
Female	Reference		Reference	
**Age groups (years)**				
20–29	0	0.990	0	0.995
30–39	6.725 (1.573–28.897)	0.008 *	6.682 (1.511–28.035)	0.009 *
40–49	1.026 (0.126–7.893)	0.837	1.010 (0.108–7.772)	0.913
50–59	3.211 (0.894–12.010)	0.075	3.145 (0.842–10.986)	0.086
≥60	Reference		Reference	
**Multiple sclerosis**				
Without	Reference		Reference	
With	1.765 (0.702–3.010)	0.245	1.628 (0.527–2.913)	0.311
**Neuromyelitis optica**				
Without	Reference		Reference	
With	3.010 (1.898–5.896)	<0.001 *	2.843 (1.625–5.113)	<0.001 *
**Acute disseminated encephalomyelitis**				
Without	Reference		Reference	
With	3.346 (2.113–6.124)	<0.001 *	3.084 (1.989–6.001)	<0.001 *
**Sarcoidosis**				
Without	Reference		Reference	
With	1.845 (0.896–3.182)	0.137	1.692 (0.533–2.873)	0.296
**SLE**				
Without	Reference		Reference	
With	2.010 (1.256–4.335)	<0.001 *	1.997 (1.154–4.237)	<0.001 *
**Behçet syndrome**				
Without	Reference		Reference	
With	0	0.985	0	0.990
**Antiphospholipid antibody syndrome**				
Without	Reference		Reference	
With	0	0.972	0	0.986
**Granulomatosis with polyangiitis**				
Without	Reference		Reference	
With	0	0.989	0	0.992
**Sicca syndrome**				
Without	Reference		Reference	
With	1.765 (0.720–2.765)	0.512	1.507 (0.562–2.631)	0.651
**CCI**	1.334 (1.112–1.509)	<0.001 *	1.218 (1.097–1.486)	<0.001 *
**Anti-TNF α use**				
Without	Reference		Reference	
With	0.674 (0.195–1.243)	0.712	0.732 (0.211–1.209)	0.564

Adjusted HR means adjusted for variables listed in the table. * indicates a significant difference between the 2 groups with/without IBD, *p* < 0.05. *CCI:* Charlson Comorbidity Index, CI: confidence interval, HR: hazard ratio, IBD: inflammatory bowel disease, NA: not available, SLE: systemic lupus *erythematosus,* TNF: tumor necrosis factor.

**Table 3:** Risk analysis for optic neuritis stratified by demographic and clinical characteristics between patients with/without IBD.

	With IBD		Without IBD		Ratio	With vs. Without (Reference)	
Stratified	Events	Rate (per 10 ^5^ PYs)	Events	Rate (per 10 ^5^ PYs)		Adjusted HR (95% CI)	*p*
**Total**	8	20.25	5	4.27	4.737	4.211 (1.352–12.978)	0.001 *
**Gender**							
Male	5	24.44	3	4.82	5.069	4.503 (1.446–13.875)	<0.001 *
Female	3	15.74	2	3.65	4.311	3.831 (1.229–11.819)	<0.001 *
**Age groups (years)**							
20–29	0	0	0	0.00	-	-	-
30–39	2	73.90	1	12.90	5.729	5.096 (1.627–15.663)	<0.001 *
40–49	1	17.60	0	0	∞	∞	0.988
50–59	3	49.03	2	11.96	4.099	3.642 (1.189–11.243)	<0.001 *
≥60	2	8.09	2	2.57	3.145	2.789 (0.842–8.512)	0.099
**Multiple sclerosis**							
Without	3	7.79	4	3.44	2.261	2.008 (0.643–6.185)	0.289
With	5	510.71	1	120.53	4.237	3.759 (1.201–11.522)	<0.001 *
**Neuromyelitis optica**							
Without	0	0	4	3.43	0	0	0.897
With	8	1050.61	1	292.72	3.589	3.202 (1.067–9.976)	<0.001 *
**Acute disseminated encephalomyelitis**							
Without	2	5.18	4	3.44	1.507	1.333 (0.415–4.086)	0.534
With	6	683.36	1	183.49	3.724	3.306 (1.048–9.975)	0.005 *
**Sarcoidosis**							
Without	4	10.28	5	4.28	2.400	2.146 (0.698–6.672)	0.349
With	4	660.00	0	0.00	∞	∞	0.880
**SLE**							
Without	3	8.03	4	3.48	2.310	2.042 (0.617–6.285)	0.297
With	5	234.00	1	53.22	4.397	3.896 (1.230–11.983)	<0.001 *
**Behçet syndrome**							
Without	8	20.38	5	4.28	4.764	4.211 (1.352–12.978)	0.001 *
With	0	0.00	0	0.00	-	-	-
**Antiphospholipid antibody syndrome**							
Without	8	20.32	5	4.28	4.754	4.211 (1.352–12.978)	0.001 *
With	0	0.00	0	0.00	-	-	-
**Granulomatosis with polyangiitis**							
Without	8	20.36	5	4.28	4.761	4.211 (1.352–12.978)	0.001 *
With	0	0.00	0	0	-	-	-
**Sicca syndrome**							
Without	4	10.53	5	4.30	2.451	2.165 (0.684–6.686)	0.275
With	4	260.00	0	0	∞	∞	0.859

Adjusted HR means adjusted for the variables listed in Table 3. * indicates a significant difference between the 2 groups with/without IBD, *p* < 0.05. *CCI:* Charlson Comorbidity Index, CI: confidence interval, HR: hazard ratio, IBD: inflammatory bowel disease, NA: not available, PYs: person-years, SLE: systemic lupus *erythematosus*. ∞ indicates infinity.

**Table 4:** Characteristics of patients with optic neuritis among the IBD cohort.

Patient	1	2	3	4	5	6	7	8
**Time to optic neuritis (years)**	0.55	1.25	2.12	4.41	5.73	8.69	9.51	11.18
**IBD type**	Ulcerative colitis	Ulcerative colitis	Crohn’s disease	Ulcerative colitis	Ulcerative colitis	Ulcerative colitis	Ulcerative colitis	Ulcerative colitis
**Sex**	Male	Female	Male	Male	Female	Male	Male	Female
**Age (years)**	47.03	59.21	34.11	56.69	84.19	72.04	59.36	35.92
**Anti-TNF α use**	No	No	No	No	Yes	No	No	Yes
**Mortality**	Survival	Survival	Survival	Survival	Mortality	Mortality	Survival	Survival

CI: confidence interval, HR: hazard ratio, IBD: inflammatory bowel disease, TNF: tumor necrosis factor.4. Discussion.

## Data Availability

Data is contained within the article or supplementary material.

## Supplementary Materials

The following are available online at https://www.mdpi.com/2077-0383/10/4/688/s1, Table S1: ICD-9-CM and definition used in this study.
