# Supplementary material for: Association between Optic Neuritis and Inflammatory Bowel Disease: A Population-Based Study
Source: J Clin Med. 2021 Feb 10;10(4):688. doi: 10.3390/jcm10040688 (PMC7916645; doi:10.3390/jcm10040688)
Supplement: Supplementary file 1 [file jcm-10-00688-s001.pdf]

Supplementary Table S1. ICD-9-CM and definition used in this study.

.	ICD-9-CM/Definition
**IBD**	
Crohn’s disease	555
Ulcerative colitis	556
**Excluding**	
Tuberculosis	017-018
Lyme disease	088.81
Syphilis	090-097, 647.0
Herpes zoster ophthalmicus	053.22, 053.29
**Optic neuritis**	Any of the listed
Optic neuritis, unspecified	377.30
Optic papillitis	377.31
Retrobulbar neuritis (acute)	377.32
Other optic neuritis	377.39
**Comorbidities**	Study period
Multiple sclerosis	340
Neuromyelitis optica	341.0
Acute disseminated encephalomyelitis	323.61
Sarcoidosis	135
SLE	710.0
Behçet syndrome	136.1, 711.2
Antiphospholipid antibody syndrome	286.53
Granulomatosis with polyangiitis	446.4
Sicca syndrome	710.2

IBD: inflammatory bowel disease, OPD: outpatient department, SLE: systemic lupus ***erythematosus,*** **TNF: tumor necrosis factor.**
